# Therapeutic effects and long-term outcomes of HMGB1-targeted therapy in rats and mice with traumatic spinal cord injury: A systematic review and meta-analysis

**DOI:** 10.3389/fnins.2022.968791

**Published:** 2022-09-07

**Authors:** Chen Deng, Li Deng, Junqiao Lv, Lin Sun

**Affiliations:** Third Hospital of Shanxi Medical University, Shanxi Bethune Hospital, Tongji Shanxi Hospital, Shanxi Academy of Medical Sciences, Taiyuan, China

**Keywords:** spinal cord injury, high mobility group box-1 (HMGB1), targeted therapy, inflammation, edema, apoptosis, functional recovery

## Abstract

**Background:**

To date, the clinical need for therapeutic methods to prevent traumatic spinal cord injury (TSCI) progression and improve functional recovery has not been met. High mobility group box-1 (HMGB1) is released by necrotic neurons or secreted by glial cells after TSCI and plays an important role in pathophysiology.

**Objective:**

The purpose of this study was to evaluate the effects of HMGB1-targeted therapy on locomotor function recovery, inflammation reduction, edema attenuation, and apoptosis reduction in rat and mouse models of TSCI.

**Methods:**

We reviewed the literature on HMGB1-targeted therapy in the treatment and prognosis of TSCI. Twelve articles were identified and analyzed from four online databases (PubMed, Web of Science, Cochrane Library and Embase) based on the Preferred Reporting Items for Systematic Reviews and Meta-Analyses (PRISMA) guidelines and strict inclusion criteria.

**Results:**

The methodological quality of the 12 articles was poor. The results of the meta-analysis showed that compared with the SCI group, the treatment group had significantly increased locomotor function scores after SCI [*n* = 159, standardized mean difference (SMD) = 2.31, 95% confidence interval (CI) (1.52, 3.10), *P* < 0.00001], and the change in locomotor function scores was significantly increased in both the drug and anti-HMGB1 Ab groups (*P* < 0.000001 and *P* < 0.000001). A subgroup analysis showed significant differences (*P* > 0.05) between the drug group [(SMD) = 1.95, 95% CI (0.95, 2.94), *P* = 0.0001] and the anti-HMGB1 Ab group [(SMD) = 2.89, 95% CI (1.66, 4.13), *P* < 0.00001]. Compared with the SCI group, HMGB1 expression was significantly diminished [*n* = 76, SMD = −2.31, 95% CI (−3.71, −0.91), *P* = 0.001], TNF-α levels were significantly reduced [*n* = 76, SMD = −2.52, 95% CI (−3.77, −1.27), *P* < 0.0001], water content was significantly reduced [*n* = 44, SMD = −3.94, 95% CI (−6.28, −1.61), *P* = 0.0009], and the number of apoptotic cells was significantly diminished [*n* = 36, SMD = −3.31, 95% CI (−6.40, −0.22), *P* = 0.04] in the spinal cord of the treatment group.

**Conclusion:**

HMGB1-targeted therapy improves locomotor function, reduces inflammation, attenuates edema, and reduces apoptosis in rats and mice with TSCI. Intrathecal injection of anti-HMGB1 Ab 0-3 h after SCI may be the most efficacious treatment.

**Systematic review registration:**

PROSPERO, identifier: CRD42022326114.

## Introduction

Spinal cord injury (SCI), as one of the most serious diseases with clinical symptoms, often leads to severe sensory and locomotor dysfunction. In recent years, its incidence has increased year by year (James, 2019), ranging from 13.1 to 163.4 per million people in developed countries and from 13.0 to 220.0 per million people in developing countries (van den Berg et al., 2010; Kang et al., 2017). Such a broad range may because of the various sampling methods and the scopes of research. Among them, non-traumatic spinal cord injury (NTSCI) accounts for a part of the proportion. Traumatic spinal cord injury (TSCI) can be divided into primary injury and secondary injury according to pathogenesis. Primary injury causes delayed damage and death to surviving adjacent cells around the lesion. Secondary injury occurs after the primary injury and is characterized by a series of biochemical events leading to further tissue loss and dysfunction through self-destructive changes in intact tissue surrounding the primary injury (Quadri et al., 2020). Secondary damage starts a few minutes after the primary injury and lasts for weeks or months, including vascular damage, inflammation, edema, apoptosis, and free radical formation, ultimately leading to the formation of cystic cavities and the maturation of glial scars (Alizadeh et al., 2019). Currently, experimental treatments for secondary injury include reducing edema and inhibiting inflammation in the acute phase of spinal cord injury; inhibiting apoptosis and glial scar evolution around the injury site in the subacute phase; regulating the formation of glial scars and matrix remodeling; and promoting the growth of axons in the chronic phase. As one of the most important therapeutic methods, regulation of the inflammatory microenvironment in the early stage of spinal cord injury has been widely studied (Hellenbrand et al., 2021).

High mobility group box-1 (HMGB1) is a highly conserved non-histone DNA binding protein. Intracellular HMGB1 plays a key role in the immune response by increasing autophagy, regulating mitochondrial function and inhibiting apoptosis (Huebener et al., 2014). Extracellular HMGB1 shows cytokine activity and acts as a typical danger-associated molecular pattern (DAMP) molecule (Wang and Zhang, 2020). After SCI, HMGB1 can be actively secreted by microglia and astrocytes or passively released by necrotic neurons (Papatheodorou et al., 2017; Yang et al., 2020). Once released, HMGB1 interacts with cell surface receptors, such as the receptor for advanced glycation end products (RAGE) and Toll-like receptors 2/4/9 (TLR-2/4/9) (Paudel et al., 2019; Sun et al., 2019), and mediates a variety of cellular responses, including promoting microglial and macrophage migration and the release of proinflammatory cytokines. It has been reported that extracellular HMGB1 can regulate the production of inflammatory factors such as interleukin-1α (IL-1α), IL-1β, IL-6, and tumor necrosis factor-α (TNF-α) and alter the expression and function of effector proteins in target cells (Man et al., 2015). In addition, inhibition of HMGB1 can reduce early edema and aquaporin-4 protein (AQP-4) expression after spinal cord injury in rats (Sun et al., 2019); AQP-4 is the main aquaporin of the central nervous system (CNS) and plays a key role in the development of edema (Halsey et al., 2018; Kitchen et al., 2020; Masterman and Ahmed, 2021).

To date, no pharmacological interventions for spinal cord injury have been approved for clinical use; however, multiple approaches to regulate HMGB1 expression have been used in preclinical animal models, such as drug intervention and anti-HMGB1 neutralizing antibody (Ab). Most of them achieved good efficacy, but some studies reported different results (Kigerl et al., 2018). The purpose of this study was to investigate the effects of HMGB1-targeted therapy on the treatment and prognosis of TSCI in rat and mouse models and to evaluate the feasibility of various therapeutic approaches.

## Materials and methods

A systematic review of the literature was conducted based on guidelines developed by the Preferred Reporting Items for Systematic Reviews and Meta-Analyses (PRISMA) (Moher et al., 2009). The study was conducted by two independent reviewers, and disagreements were resolved by discussion with a third reviewer.

### Literature search

Published papers analyzing the therapeutic and prognostic effects of HMGB1 in rat and mouse spinal cord injury models were identified through a search of the PubMed, Web of Science Cochrane Library and Embase electronic databases. Key words included “spinal cord injury,” “SCI,” “high mobility group box protein 1” and “HMGB1.” As an example, the details of the PUBMED database search strategy are as follows: ‘{“Spinal Cord Injuries”(MeSH Terms) OR [“SCI”(Title/Abstract) OR “spinal cord injury”(Title/Abstract)]} AND [“HMGB1 Protein”(MeSH Terms) OR “HMGB1”(Title/Abstract) OR “high mobility group box protein 1”(Title/Abstract)]'. Studies were screened by title and abstract according to the inclusion and exclusion criteria listed, and duplicated studies were removed. The shortlisted studies were independently reviewed again, and the full text was read to select the final research choice for subsequent analysis.

### Inclusion and exclusion criteria

#### Inclusion criteria

(1) Population: experimental rat or mouse studies involving traumatic SCI of any age or sex, including contusion, crush and compression injury;

(2) Intervention: HMGB1 intervention with no limitations on the method of administration, formulation or dosage;

(3) Comparator: any type of placebo control group, such as DMSO (dimethyl sulfoxide), physiological saline, PBS, IgG2a antibodies (isotype control) or no treatment;

(4) Outcome: (a)locomotor functional evaluation: the Basso, Beattie, and Bresnahan (BBB) rating scale or the Basso Mice Scale (BMS) rating scale; (b) biochemical examinations: HMGB1 expression, TNF-α levels, spinal cord water content and apoptotic cell count;

(5) Study design: controlled studies assessing the *in vivo* administration of HMGB1-targeted therapy to rats or mice with SCI; original study published in English; no publication date or publication status restrictions were imposed.

#### Exclusion criteria

(1) Studies that were not controlled studies, such as reviews, systematic reviews, case reports or meetings.

(2) Substandard animal models (chronic constriction injury model; spinal cord ischemia–reperfusion injury model; models other than in rats or mice).

(3) Clinical and *in vitro* studies.

(4) No relevant outcomes reported.

(5) Repeated publications.

### Assessment of risk of bias in included studies

The risk of bias for the included studies was assessed using the Systematic Review Center for Laboratory Animal Experimentation (SYRCLE) risk of bias tool for animal studies, which was adapted from Cochrane's risk of bias tool (Hooijmans et al., 2014). The risks of bias were assessed by two independent reviewers (CD and LD) for each study. The risk of bias was assessed as a low or high risk of bias, and “unclear risk” indicated that the risk of bias was not clear.

### Data extraction

Two reviewers independently extracted details from the studies included in the meta-analysis. It included the first author, date of publication, animal strain, weight, sex, number of animals in each group, method used to induce SCI, SCI level, type of intervention, timing of intervention, daily dose of intervention, follow-up time frame after SCI, and outcomes of significance to SCI. The mean standard deviation (SD) of experimental results and the number of animals in the treatment group and SCI group were extracted from these data for meta-analysis. According to the observation, the first analysis of locomotor function is usually performed within 48 h, which may be the reason for the score of 0; in this case, the measurement is not considered to be the first time. If locomotor function was evaluated more than once, SD changes were used for a meta-analysis. If the biochemical examinations were performed at different times, the last outcome indicators that were measured after SCI were adopted. The last measurements were taken no later than 14 days post-SCI. If there were any controversies, we settled the problem by discussion and with the help of a third reviewer.

All studies included the SCI group and treatment groups. However, some of the results were presented in the form of a graph rather than an exact report of the raw data they obtained, which meant that the actual numbers had to be estimated from the data extracted from the graph. GetData Graph Digitizer 2.24 software was used to estimate numerical values from the graphs, and the study was excluded if the required data were not presented or obtainable.

### Data synthesis

The data were then synthesized through a meta-analysis. The main objective is to provide a more accurate estimate of the effect size of the treatment. According to different treatment methods, 12 studies including locomotor function recovery data were divided into the following two groups for subgroup analysis: anti-HMGB1 Ab group and drug group. We also performed a meta-analysis on the reduction of HMGB1 and TNF-α levels, the attenuation of edema, and the reduction of apoptosis after the various treatments.

### Outcome measures

The main outcome measures were locomotor functional evaluation (the BBB rating scale and the BMS rating scale) and biochemical examinations, including HMGB1 expression, TNF-α level, spinal cord water content and apoptotic cell count.

### Statistical analysis

Meta-analysis was performed using Review Manager version 5.3 (Cochrane Collaboration). Data from the treatment group were compared with those from the spinal cord injury group. Data were pooled if at least three studies reported results, and continuous variables were expressed as the mean difference (MD) or standardized mean difference (SMD), both with 95% CI. If the unit of measurement was consistent, MD was used to evaluate the effect size; SMD was used to evaluate the effect size when the measurement units were different. A chi-square test was used for heterogeneity: *P* < 0.1 represents heterogeneity, and *P* > 0.1 indicates no heterogeneity. I2 statistics were also used to assess heterogeneity: 0% ≤ I2 < 25% indicated no heterogeneity; 25% ≤ I2 < 50% indicated low heterogeneity; 50% ≤ I2 < 75% showed moderate heterogeneity; and 75% ≤ I2 revealed high heterogeneity. When heterogeneity between studies was low, the fixed effects model was used to estimate the combined effect size; otherwise, the random effects model was used. P <0.05 was considered statistically significant, and publication bias was determined by funnel plot.

## Results

### Study selection

**Figure 1:**
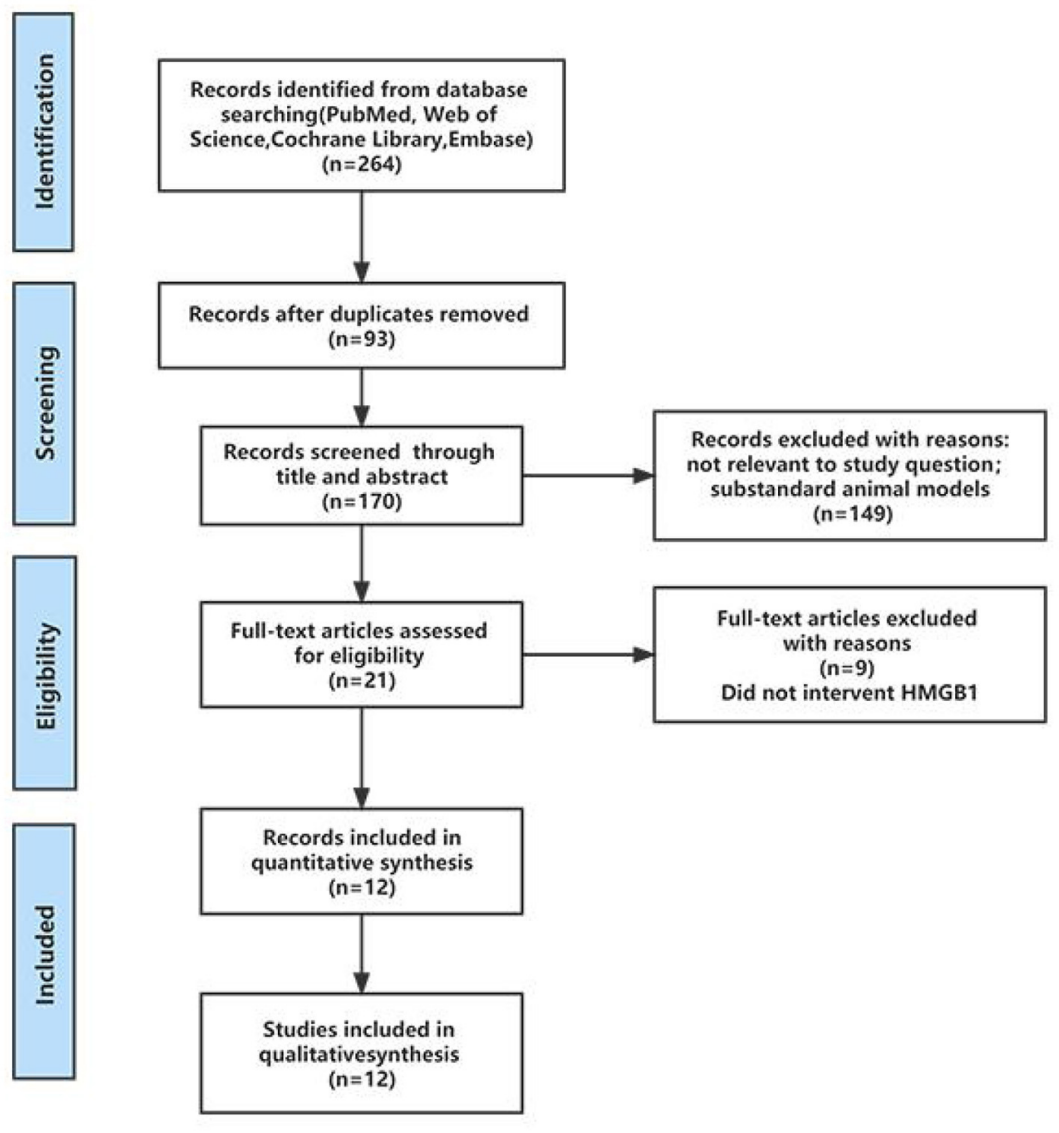
PRISMA flow diagram to demonstrate the screening process for included studies in this systematic review.

A total of 264 results were retrieved from four databases: PubMed, Web of Science, Cochrane Library and EMBASE. After deleting duplicate versions, 170 articles were initially screened. According to previously defined inclusion and exclusion criteria, 149 studies were deleted through titles and abstracts, leaving only 21 studies eligible for study and requiring full reading. After full-text screening, 9 studies were excluded because there was no intervention on HMGB1, so 12 studies were included for further analysis. One article included two treatment groups compared with the same control group, and both studies were included in this study. Therefore, 13 studies were included in this study, which contained 12 articles. The process of the literature identification and ranking strategy is shown in the PRISMA diagram (Figure 1).

### Study characteristics

The characteristics of the studies included in this systematic review are shown in Table 1. Most of the 12 articles that met the inclusion criteria were from China, while the remaining three were from Japan (Uezono et al., 2018; Nakajo et al., 2019; Zhu et al., 2021) and one was from the US (Kigerl et al., 2018). All of the studies were randomized controlled trials. Seven studies used SD rats with weights ranging from 180 to 270 g; four studies used C57BL/6J mice ranging in weight from 18 to 25 g; and only one study (Uezono et al., 2018) used non-obese diabetic severe combined immunodeficient (NOD-SCID) mice. The total sample size of the study ranged from 15 to 296, with an average sample size of 79. Four studies used male animals, six used female animals, and the other studies did not report gender information. Eleven studies used contusion injury, and two other studies (Fan et al., 2020; Wu et al., 2021) used compression injury. In all of the studies, SCI was performed at T10 except one study at T8 (Fan et al., 2020) and another at T9 (Kigerl et al., 2018).

**Table 1:** Characteristics of the included studies.

**Study ID**	**Country**	**Animals**	**Level of SCI**	**Injury**	**Group design**	**Therpay**	**Follow up time after SCI**	**Outcome measures**
**Rat**								
Yang et al. (2013)	China	Adult Sprague-Dawley rats (250–300 g)	T10	Allen's Weight-Dropping method (10 g*25 mm)	Sham (40) sham+HBO (40) SCI (40) SCI +HBO (40)	HBO(1 h,8~10 L/min, 95%,once daily)	1d, 3d, 7d, 14d	HMGB1, NF-κB expression WB/RT-PCR/IHC; locomotor function.
Kang et al. (2015)	China	Adult Sprague-Dawley rats (250–300 g)	T10	Allen's Weight-Dropping method (10 g*25 mm)	Sham (30) sham+HBO (30) SCI (30) SCI +HBO (30)	HBO(1 h,8~10 L/min, 95%,once daily)	1d, 2d, 3d, 7d, 14d	HMGB1, TLR4 expression WB/RT-PCR/IHC; HMGB1, NF-κB, IL-1β, TNF-α level ELISA; locomotor function.
Bi et al. (2017)	China	Adult male Sprague-Dawley rats (180–220 g)	T10	Modified Allen's Weight-Dropping method (8 g*40 mm)	Sham (8) SCI (8) SCI+MPSS (8) SCI+Shi(100) (8)	MPSS(100 mg/kg, Immediately); Shinkonin (100 mg/kg, Immediately)	1d, 2d, 3d	HMGB1, TLR4, NF-κB expression WB/RT-PCR; HE staining; IL-1β,IL-6,TNF-α level ELISA; water content; Tunel staining; caspase-3 expression WB; locomotor function.
Sun et al. (2019)	China	Adult female Sprague-Dawley rats	T10	Modified Allen's Weight-Dropping method (10 g*25 mm)	Sham (66) SCI (66) SCI+EP (66) SCI+GL (66)	EP (50 mg/kg, once daily) GL(100 mg/kg, once daily)	12h, 1d, 3d, 7d, 10d, 14d	HMGB1, GFAP, AQP-4, expression WB/IHC; HMGB1 level ELISA; TLR4/MyD88 pathway activation; water content; oedema via MRI; locomotor function.
Fan et al. (2020)	China	Adult male Sprague- Dawley rats (250 g)	T8	Modified Tetzlaff spinal cord lateral crush model (Last 20 s)	Sham (37) SCI (37) SCI+GL (37) SCI+ FPS-ZM1 (37)	GL (10 mg/kg, immediately,14 days after SCI) FPS-ZM1 (1 mg/kg, immediately,14 days after SCI)	1d, 3d, 7d, 10d, 14d, 21d	iNOS, IL-12, CD86, TNF-α expression RT-PCR; rage iNOS iba-1expression IF; Nissl staining; lesion area; neuronal survival; behavioral evaluation
Chen et al. (2021)	China	Adult female Sprague-Dawley rats (270 g)	T10	Contusion injury using a weight drop device (10 g*12.5 mm)	Control (8) Anti-HMGB1 (8)	anti-HMGB1 Ab (50 ng/μl, 1 μl, Immediately)	1w, 2w, 3w, 4w, 5w, 6w	TNF-α IFN-γ IL-1α IL-6 IL-17 level LiquiChip assay; HE staining; Nissl staining; microglia polarization; locomotor function
Wu et al. (2021)	China	Adult male Sprague-Dawley rats (200–220 g)	T10	Compression injury using an aneurysm clip (Last 10 s)	Sham (18) SCI (18) SCI+GA (18)	GA (100 mg/kg, once daily)	3d	HMGB1, TNF-a, IL-1b, IL-6 expression WB/RT-PCR/IHC; HE staining; microglia expression; p38/JNK pathway activation
**Mice**								
Zhang et al. (2014)	China	Male C57BL/6J mice (20–25 g)	T10	Contusion injury using a Infinite Horizons Impactor (60 kydn)	Sham(6) Control(6) HG(6) ZnPPIX(6)	Higenamine (HG,10 mg/kg, immediately), ZnPPIX (10 mg/kg, immediately)	1d, 3d, 7d, 14d, 28d, 42d	HMGB1, IFN-γ, TNF-α, IL-4, IL-10 expression WB; macrophages expression; GAP-43, NF-H IHC; HMGB1 ELISA; locomotor function.
Kigerl et al. (2018)	US	Female C57BL/6 mice	T9	Contusion injury using a Infinite Horizons injury device (70 kydn)	Sham (6) Control (6) anti-HMGB1 mAb (6)	anti-HMGB1 mAb (50 μg/day, 1 day prior to SCI,7 days)	1d, 3d, 7d, 14d, 21d, 28d, 35d, 42d	HMGB1 expression WB/RT-PCR/IF; microglia/macrophages expression; lesion area; locomotor function
Uezono et al. (2018)	Japan	female (NOD-SCID) mice (18–22 g)	T10	Contusion injury using a Infinite Horizon Impactor (70 kydn)	Non-treatment (16) Transplantation alone (16) anti-HMGB1 mAb alone (12)	anti-HMGB1 mAb (8 mg/kg, 5 min and 6 h after SCI) hiPSC-NSCs (2.5*10^5^μl^−1^,2μl,7d after SCI)	1d, 3d, 7d, 14d, 28d, 42d	HMGB1, IFN-γ, TNF-α, IL-4, IL-10 expression WB; GFAP IHC; tunel staining; lesion area; neuronal survival; behavioral analysis electrophysiology; Evans Blue dye extravasation; water content
Nakajo et al. (2019)	Japan	Female C57BL/6J mice (18–22 g)	T10	Contusion injury using a Infinite Horizon Impactor (70 kydn)	Sham (5) Control (5) anti-HMGB1 mAb (5)	anti-HMGB1 mAb (8 mg/kg, 0 or 3 or 6 or 9 or 12 h after SCI.)	1w, 2w, 3w, 4w, 5w, 6w−12w	TNF-a, IL-1b, IL-6, MMP-2/9 expression RT-PCR; tunel staining; lesion area; neuronal survival; behavioral analysis; Evans Blue dye extravasation; water content
Zhu et al. (2021)	Japan	Female C57BL/6J mice (18–22 g)	T10	Contusion injury using a Infinite Horizon Impactor (70 kydn)	Control (10) Epo B (10) anti-HMGB1 mAb (10) Combination (10)	anti-HMGB1 mAb (8 mg/kg, 5 min after SCI) Epo B (3 mg/kg, 1d, 15d after SCI)	0d, 7d, 14d, 28d, 42d, 49d, 56d	GFAP expression IF; glial scar formation; neuronal survival; behavioral analysis

NF-κB, nuclear factor-kappa B; GFAP, glial fibrillary acidic protein; GAP-43, growth-associated protein-43; NF-H, neurofilament-H; MyD88, myeloid differentiation primary response 88; RT-PCR, real-time polymerase chain reaction; WB, Western blot; HE, haematoxylin–eosin; IF, immunofluorescence; IHC, immunohistochemistry.

In terms of treatment, inhibiting the role of HMGB1 *in vivo* was chosen by all studies. Five of the studies used an anti-HMGB1 Ab (Kigerl et al., 2018; Uezono et al., 2018; Nakajo et al., 2019; Chen et al., 2021; Zhu et al., 2021), most of which was injected within 0-6 hours after injury at a dose of 8 mg/kg, which significantly improved locomotor function recovery. However, in one study (Kigerl et al., 2018), a dose of 50 μg per day injected 1 day before injury and last 7 days had no significant therapeutic effect. In two studies (Yang et al., 2013; Kang et al., 2015), hyperbaric oxygen (HBO) was used to downregulate HMGB1 expression, with an oxygen flow rate of 8-10 L/min and an oxygen concentration of 95%, once a day for 1 h. Three studies (Sun et al., 2019; Fan et al., 2020; Wu et al., 2021) used glycyrrhizin (GL) at doses of 10 or 100 mg/kg, injected immediately after injury, once daily; other studies used shinkonin (Bi et al., 2017) (100 mg/kg), ethyl pyruvate (Sun et al., 2019) (EP) (50 mg/kg) or higenamine (Zhang et al., 2014) (HG) (10 mg/kg), which significantly inhibited HMGB1 expression after spinal cord injury.

Most studies reported results using locomotor function assessment and biochemical analysis. BBB scores were used to assess locomotor function 12 h-42 days after injury in rats. BMS scores were used to assess locomotor function 1-12 weeks after injury in mice. Most studies have examined the expression of inflammatory mediators such as HMGB1 and TNF-α after spinal cord injury. Some studies have also reported the degree of spinal edema and the number of apoptotic cells to detect the characteristics of spinal cord injury. In addition, electrophysiological examination (Uezono et al., 2018) and Evans blue dye extravasation (Uezono et al., 2018; Nakajo et al., 2019) were reported in some studies, which were not included in the meta-analysis due to the small number of studies.

### Bias analysis of included studies

**Figure 2:**
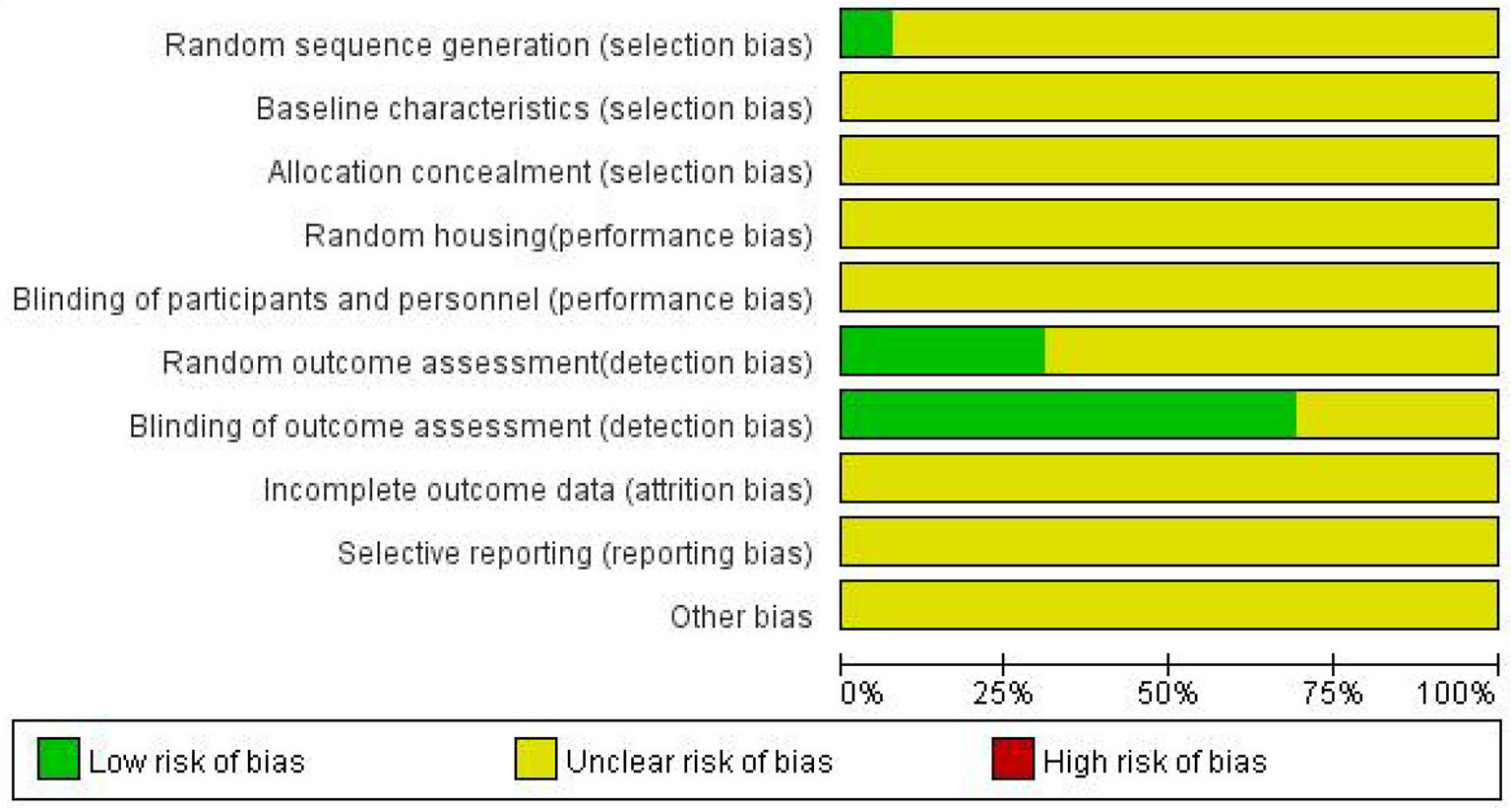
Risk of bias.

The risks of bias for all 13 studies included in the 12 articles are shown in Figure 2. The process assessed studies across 10 areas, and from the information provided, they were classified as low risk, high risk or unclear risk. Overall, the methodological quality of the included studies was not high. No study described baseline characteristics, allocation concealment, random housing, blinding of participants and personnel, incomplete outcome data, selective reporting or other bias. Nine studies described blinding of outcome assessment. Random outcome assessments were reported in four studies. Only one study (Yang, et al., 2013) reported random sequence generation using the randomization table method.3.4 Meta-analysis.

#### Assessment of locomotor function improvement by inhibiting HMGB1

**Figure 3:**
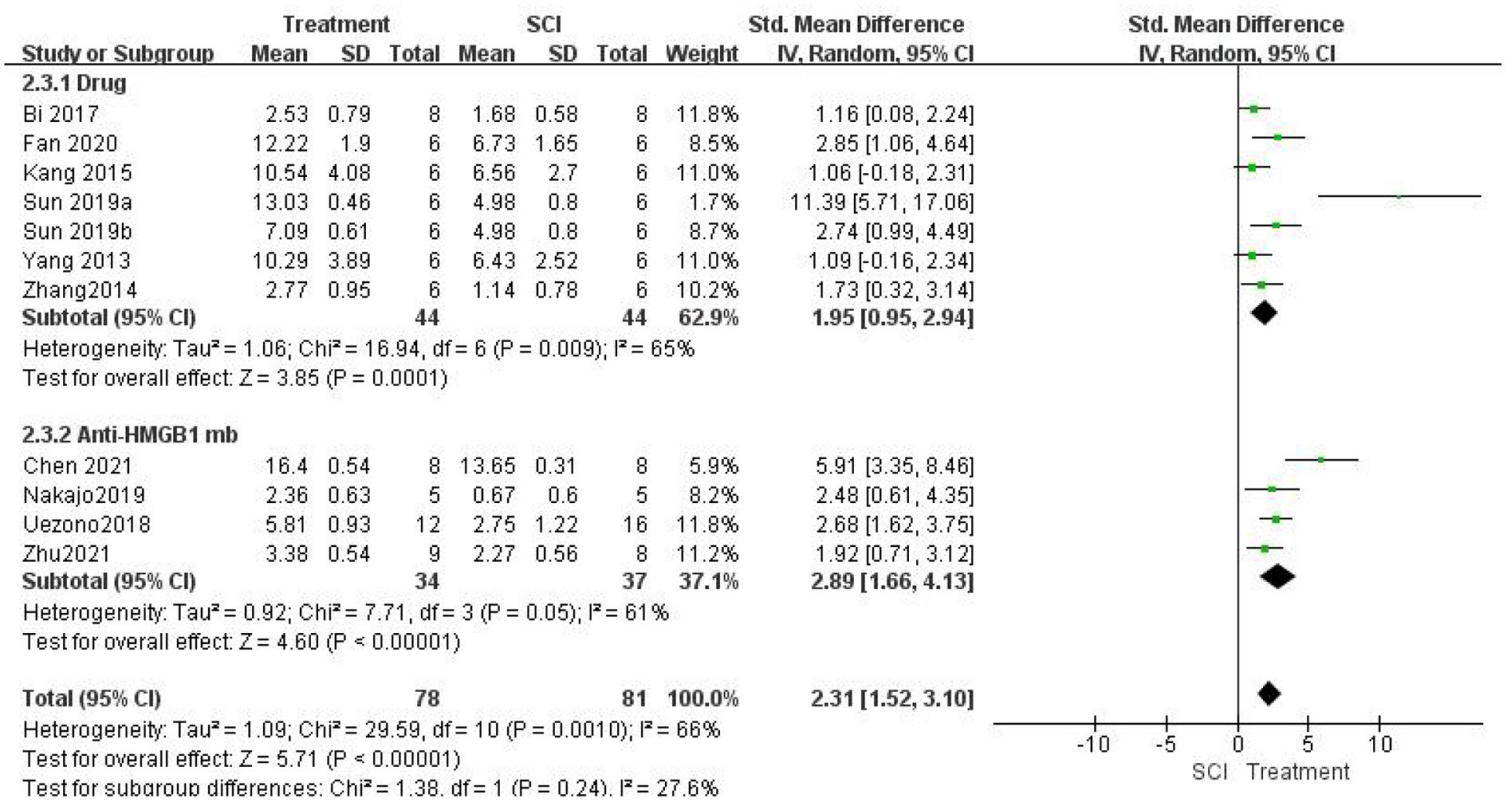
Forest plot for the effects of HMGB1 intervention on locomotor function scores in SCI (random-effects model). Green dots indicate weighted effect sizes for 11 treatment regimens and error bars indicate 95% confidence intervals for each outcome. Heterogeneity of the study is indicated by the I2 statistic. *p* < 0.001 indicates statistical significance.

A meta-analysis of locomotor function score data from 11 studies showed that inhibiting the role of HMGB1 *in vivo* significantly promoted locomotor function recovery. Inhibiting the role of HMGB1 *in vivo* significantly increased locomotor function scores [11 studies, *n* = 159, SMD = 2.31, 95% CI (1.52–3.10), *P* < 0.00001; Figure 3] in a random-effects model because of the moderate heterogeneity (I2 = 66%, *P* = 0.001).

Then, we performed a subgroup analysis. Seven studies (Yang et al., 2013; Zhang et al., 2014; Kang et al., 2015; Bi et al., 2017; Sun et al., 2019; Fan et al., 2020) used different drug interventions in the drug group and found that the change in locomotor function scores in the treatment group was significantly higher than that in the SCI group using a random-effects model [seven studies, *n* = 88, SMD = 1.95, 95% CI (0.95–2.94), *P* = 0.0001; Figure 3]. There was moderate heterogeneity in this group of studies (I2 = 65%, *P* = 0.009). Four studies (Uezono et al., 2018; Nakajo et al., 2019; Chen et al., 2021; Zhu et al., 2021) used anti-HMGB1 Ab and found that the locomotor function scores in the treatment group were significantly higher than those in the SCI group using a random-effects model [four studies, *n* = 71, SMD = 2.89, 95% CI (1.66–4.13), *P* < 0.00001; Figure 3]. The heterogeneity was moderate in this group of studies (I2 = 61%, *P* = 0.05). In addition, there were significant differences between subgroups (I2 = 27.6%, *P* = 0.24).

Locomotor function recovery was measured in one study (Kigerl et al., 2018) with BMS scores, and no differences were found between the control groups and SCI mice treated with anti-HMGB1 Ab (*P* > 0.05). One study (Sun et al., 2019) also measured locomotor function recovery using the inclined plane score. Significant increases in the mean inclined plane test results were found compared with the SCI group (*P* < 0.05). Another study (Fan et al., 2020) used the rump height index (RHI) assay to assess improvement in locomotor function. The results indicated that the RHI value increased significantly compared with that of the SCI group (*P* < 0.05).

#### Assessment of the anti-inflammatory effect of inhibiting HMGB1

**Figure 4:**
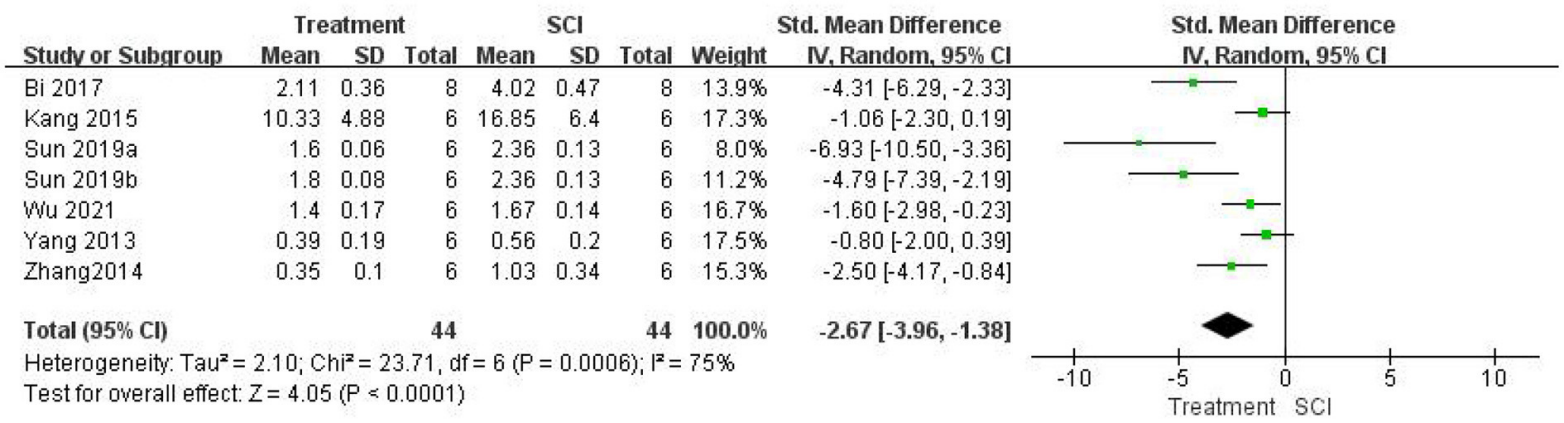
Forest plot for the effects of HMGB1 intervention on HMGB1 expression in SCI (random-effects model).

First, we evaluated whether the treatment in this study significantly inhibited the role of HMGB1 *in vivo*. Five studies (Kigerl et al., 2018; Uezono et al., 2018; Nakajo et al., 2019; Chen et al., 2021; Zhu et al., 2021) used HMGB1 neutralizing Abs and did not examine HMGB1 expression. Seven studies (Yang et al., 2013; Zhang et al., 2014; Kang et al., 2015; Bi et al., 2017; Sun et al., 2019; Wu et al., 2021) measured HMGB1 expression levels after SCI. HMGB1 levels were significantly lower in the treatment group than in the SCI groups using a random-effects model [seven studies, *n* = 88, SMD = −2.67, 95% CI (−3.96 to −1.38), *P* < 0.0001; Figure 4]. Heterogeneity was significantly high in this group of studies (I2 = 75%, *P* = 0.0006).

**Figure 5:**
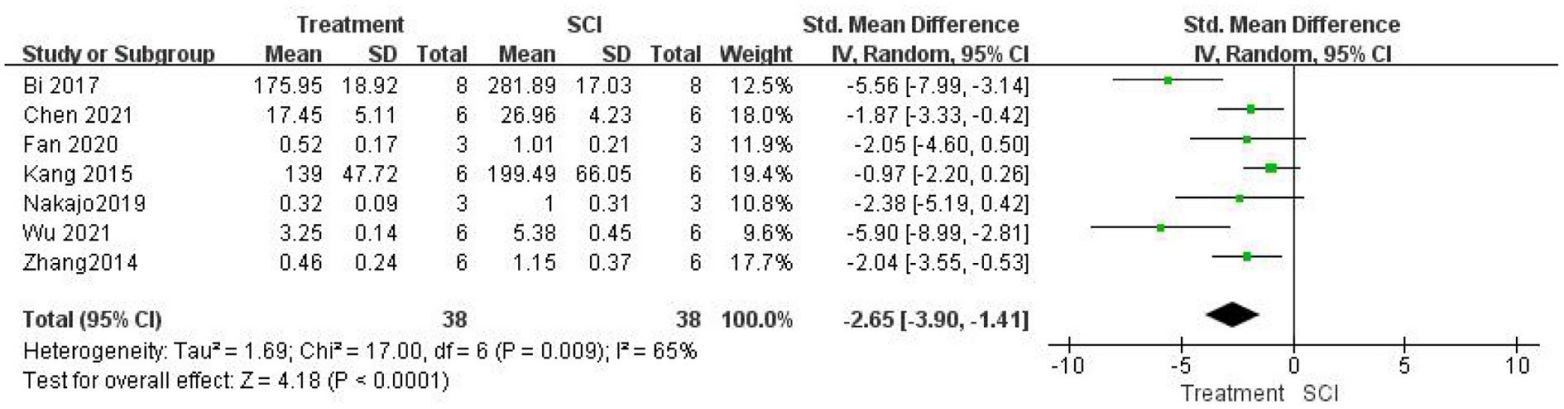
Forest plot for the effects of HMGB1 intervention on TNF-α levels in SCI (random-effects model).

Seven studies (Zhang et al., 2014; Kang et al., 2015; Bi et al., 2017; Nakajo et al., 2019; Fan et al., 2020; Chen et al., 2021; Wu et al., 2021) measured TNF-α levels after SCI and found that TNF-α levels were significantly lower in the treatment group than in the SCI groups using a random-effects model [seven studies, *n* = 76, SMD = −2.65, 95% CI (−3.90 to −1.41), *P* < 0.0001; Figure 5]. Heterogeneity was moderate in this group of studies (I2 = 65%, *P* = 0.0009).

#### Assessment of the attenuation of edema by inhibiting HMGB1

**Figure 6:**
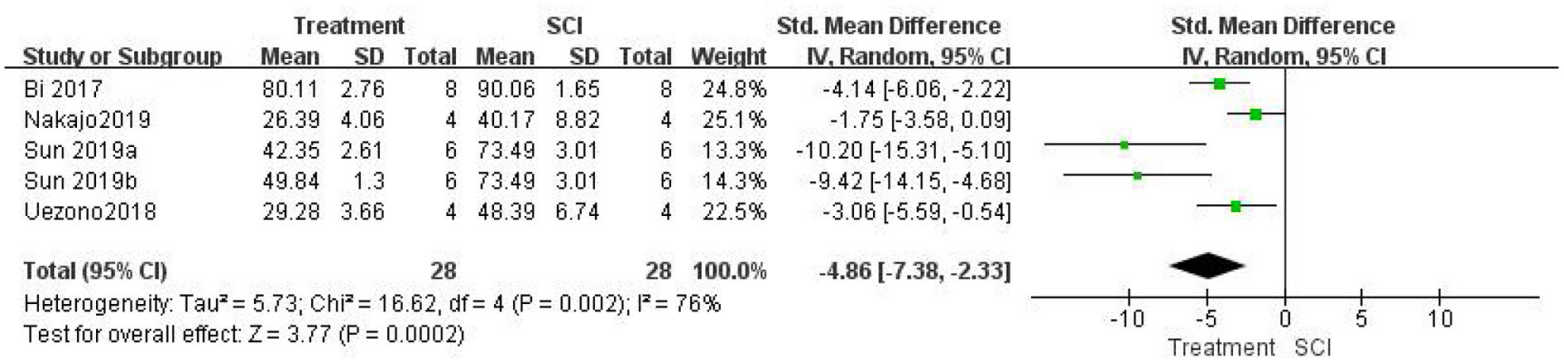
Forest plot for the effects of HMGB1 intervention on spinal cord water content in SCI (random-effects model).

Five studies (Bi et al., 2017; Uezono et al., 2018; Nakajo et al., 2019; Sun et al., 2019) examined spinal cord water content. A meta-analysis showed that inhibiting the role of HMGB1 *in vivo* could significantly reduce spinal cord edema. Water content was significantly lower in the treatment group than in the SCI groups [five studies, *n* = 56, SMD = −4.86, 95% CI (−7.38 to −2.33), *P* = 0.0002; Figure 6] using a random-effects model because the degree of heterogeneity was high (I2 = 76%, *P* = 0.002).

#### Assessment of the reduction in apoptosis after inhibiting HMGB1

**Figure 7:**
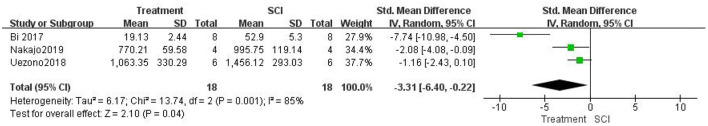
Forest plot for the effects of HMGB1 intervention on apoptosis in SCI (random-effects model).

Three studies (Bi et al., 2017; Uezono et al., 2018; Nakajo et al., 2019) measured the number of apoptotic cells after SCI and found that the number of apoptotic cells was significantly lower in the treatment group than in the SCI groups using a random-effects model (three studies, *n* = 36, SMD = −3.31, 95% CI (−6.40– −0.22), *P* = 0.04; Figure 7]. Heterogeneity was significantly high in this group of studies (I2 = 85%, *P* = 0.001).

### Publication bias

**Figure 8:**
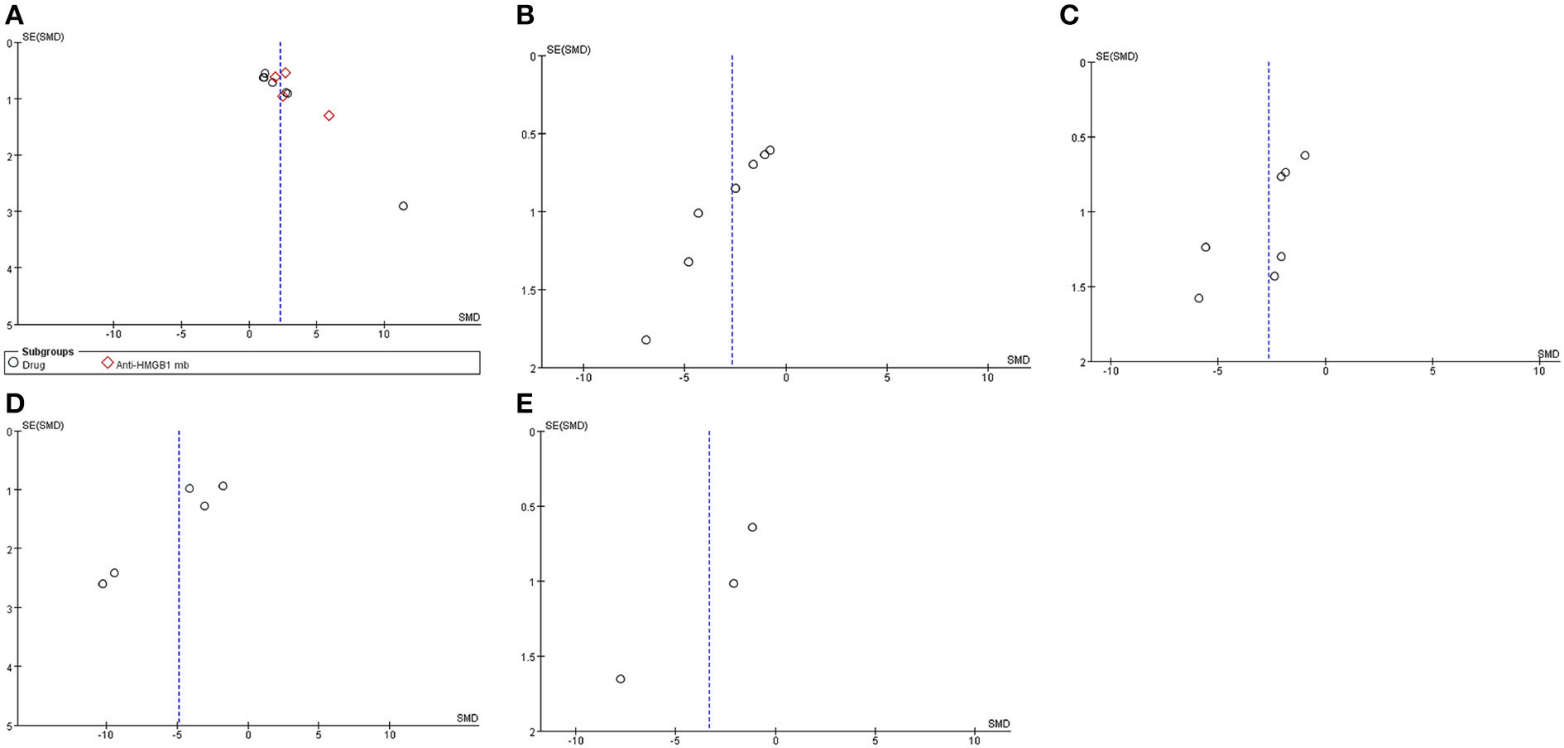
Asymmetries found in the funnel plots indicated the possibility of publication bias. Funnel plots of publication bias for locomotor function score **(A)**, HMGB1 levels **(B)**, TNF-α levels **(C)**, water content **(D)**, and apoptosis **(E)**.

Funnel plots of publication bias for locomotor function scores were assessed (Figure 8). The asymmetries found in the funnel plots indicated the possibility of publication bias.

## Discussion

### Summary of evidence

In this study, we first systematically reviewed the recently published literature on the therapeutic and prognostic role of HMGB1-targeted therapy in TSCI. The search identified 170 studies, which were eventually narrowed down to 12 by applying our inclusion/exclusion criteria. After reading the full text, the outcome characteristics were extracted and analyzed, and finally, a meta-analysis was conducted to calculate an accurate estimate of the effect size for each treatment.

All studies investigated the efficacy of targeting HMGB1 in SCI treatment. Seven studies (Yang et al., 2013; Zhang et al., 2014; Kang et al., 2015; Bi et al., 2017; Sun et al., 2019; Wu et al., 2021) selected different drugs to inhibit HMGB1 expression. Moreover, there were variations of the timing and protocol of the intervention in the above studies (Table 1).

Glycyrrhizin (GL) is a natural anti-inflammatory found in licorice root that is able to inhibit HGMB1 and its cytokine-like signaling, ethyl pyruvate (EP) has also been shown to be a neuroprotective therapeutic agent, inhibiting neuroinflammation and promoting spinal cord repair. Sun et al. (Sun et al., 2019) used EP or GL *via* an intraperitoneal injection to inhibit HMGB1. Rats received a 50 mg/kg dose of EP (diluted in 0.9% saline) or a 100 mg/kg dose of GL (diluted in 0.9% saline) *via* an intraperitoneal injection (i.p.) immediately after SCI, and then continued to receive this injection daily after the injury. The results showed that EP and GL inhibited HMGB1 expression in the spinal cord and HMGB1 levels in the serum of SCI rats. Inhibition of HMGB1 can improve locomotor function and reduce spinal water content and AQP-4 overexpression in rats with spinal cord injury. Furthermore, HMGB1 inhibition also repressed the activation of the TLR-4/myeloid differentiation primary response gene 88 (Myd 88)/nuclear factor-kappa B (NF-κB) signaling pathway. Two other studies (Fan et al., 2020; Wu et al., 2021) also used GL, and both of them significantly reduced the expression levels of HMGB1 and inflammatory factors. Fan et al. (2020) showed that a 10 mg/kg dose of GL in saline administered daily *via* i.p. for 14 days after SCI and the first injection immediately after SCI inhibited HMGB1 expression, decreased the number of proinflammatory macrophages/microglia after SCI through the RAGE/NF-κB pathway, reduced neuronal loss and demyelination and improved functional recovery. However, Wu et al. (2021) used different doses. In this study, GL was diluted in dimethyl sulfoxide and 20% sulfobutylether-b-cyclodextrin in 0.9% saline. Rats received 100 mg/kg GL by i.p. immediately after injury, and then daily for 3 days. The results demonstrated that GL could inhibit HMGB1 expression through the P38/Jun N-terminal kinase (JNK) pathway, thus alleviating the inflammatory response after SCI.

Shikonin is the major bioactive component extracted from the roots of Lithospermum erythrorhizon. Recent studies have shown that shikonin is an effective inhibitor of protein–protein interactions with multiple targets in both the intracellular and extracellular compartments, and exhibits a variety of biological activities related to cancer treatment, inflammation and wound healing (Chen et al., 2002; Guo et al., 2019a). Its anti-inflammatory effect may be related to the downregulation of HMGB1 expression (Yang et al., 2014; Bi et al., 2017). One study (Bi et al., 2017) used 100 mg/kg shikonin administered by intraperitoneal injection 30 min after SCI to downregulate HMGB1 levels and showed that shikonin could promote the recovery of locomotor function, suppress apoptosis and inhibit spinal cord edema *via* inactivation of the HMGB1/TLR4/NF-κB signaling pathway in an SCI model in rats.

HG, an active ingredient of Aconiti Lateralis Radix Praeparata, has been traditionally used as an anti-inflammatory agent in oriental countries (Zhang et al., 2017). A study has shown that HG plays protective roles in brain injury by inducing the upregulation of HO-1 to reduce the level of HMGB1 (Ha et al., 2012). Among the seven studies, one study (Zhang et al., 2014) used HG as an anti-inflammatory agent. The results showed that 10 mg/kg HG *via i*.p. just after SCI increased the expression of IL-4 and IL-10 and promoted M2 macrophage activation, reduced HMGB1 expression dependent on HO-1 induction and then promoted locomotor function after SCI.

In recent years, HBO therapy has received increasing attention in reducing the inflammatory response and apoptosis in SCI (Meng et al., 2019; Ying et al., 2019; Zhou et al., 2019b). HBO intervention was used in two studies (Yang et al., 2013; Kang et al., 2015) with the same timing and protocol. During HBO intervention, continuous ventilation was maintained for 1 h with oxygen flow of 8–10 L/min and chamber oxygen concentration exceeding 95%. The intervention was managed once daily. The results demonstrated that HBO intervention could reduce the secondary damage of SCI caused by inflammatory responses by decreasing the expression of HMGB1 and TNF-α and promoting the recovery of locomotor function by downregulating HMGB1/TLR4/NF-KB pathway.

Five studies (Kigerl et al., 2018; Uezono et al., 2018; Nakajo et al., 2019; Chen et al., 2021; Zhu et al., 2021) using anti-HMGB1 Ab were included in this study. Three of the studies (Uezono et al., 2018; Nakajo et al., 2019; Zhu et al., 2021) improved the recovery of locomotor function with intraperitoneal injections of anti-HMGB1 monoclonal antibody (mAb) (IgG2a subclass, 8 mg/kg). The mAb recognition epitopes were identified by using synthetic overlapping peptides of 15 amino acids in length derived from the human HMGB1 sequence. The clone (#10–22, subclass IgG2a) recognizing the C-terminal sequence of the HMGB1 molecule (DEDEEEE) was used for the experiments (Liu et al., 2007). Moreover, it is important to note that the timing of mAb injection varies. Uezono et al. (2018) opted two injections 5 min and 6 h after injury. Zhu et al. (2021) selected a single injection 5 min after spinal cord injury. The results showed that both interventions significantly improved motor function recovery after SCI in rats, but two injections improved locomotor function better than a single injection (2.68>1.92, Figure 3). However, from the perspective of clinical application, 5 min after spinal cord injury is not practical. Nakajo et al. (2019) chosed to inject mAb at 0 h, 3 h, 6 h, 9 h and 12 h after SCI to test the therapeutic time window of mAb administration. The results showed that there was no significant difference in functional recovery between the 0 h and 3 h administered groups, while injection at 6 h had a poor effect, and injection at 9 and 12 h had no therapeutic effect.

Kigerl et al. (2018) also received i.p. of anti-HMGB1 mAb, but the improvement in locomotor function was not statistically significant. There are several possible reasons. First, the mAb recognition epitopes were different. Overlapping 18–amino acid peptides covering the entire HMGB1 sequence were synthesized for epitope mapping. Clone 1 bound to a region between amino acids 53 and 63 and clone 2 bound to amino acids 67–78 of the protein, both in the A box region of HMGB1 (Qin et al., 2006). Second, the timing and protocol of the injection are different. Ab was injected daily *via* i.p. injection (50 μg/day) starting 1 day prior to SCI and continuing for 7 days. Under this administration, intraperitoneal route of administration (50 μg/day) may not allow sufficient concentration of blocking antibodies to accumulate in tissue at the site of injury and adjacent to the intact spinal cord. Interestingly, in another study (Chen et al., 2021), although they used polyclonal antibodies, which are less specific than mAb, at a dose of 1 μl (50 ng/μl), intrathecal injection of anti-HMGB1 Ab immediately after SCI improved locomotor function more significantly than i.p. (5.91 > 2.68, Figure 3). Therefore, a more direct route of delivery may improve access and binding efficiency. Intrathecal injections are a better option, bypassing the blood–spinal barrier and ensuring sufficient concentrations of antibodies can accumulate at the site of spinal cord injury. In addition, subgroup analysis showed that there was a greater improvement in locomotor function in the anti-HMGB1 Ab group than in the drug group (2.89 > 1.95, I2 = 27.6%, *P* = 0.24); thus, immediate intrathecal injection after SCI may be a more efficacious treatment. It has been reported that neuroprotective factors have a longer treatment window for spinal cord injury in primate models than in rodent models (Nishimura et al., 2014). From the perspective of clinical application, 0–3 hours after spinal cord injury may be a more practical choice.

### Possible mechanism of the effect of HMGB1-targeted therapy in TSCI

We found that HMGB1-targeted therapy can improve functional recovery after spinal cord injury in a variety of ways (Nishibori et al., 2019; Zhou et al., 2019a; Chen et al., 2021). This is mostly through the following three key modalities: reduce the release of inflammatory factors and inhibit inflammation in the early stage of spinal cord injury; reduce the expression of AQP-4 and attenuate spinal cord edema; and inhibit cell apoptosis.

Traumatic spinal cord injury (TSCI) can lead to a systemic inflammatory response, that is, an increase in immune cells and proinflammatory mediators, resulting in the persistence of an inflammatory microenvironment that ultimately leads to organ dysfunction (Sun et al., 2016). The local inflammatory microenvironment in the injured spinal cord includes necrotic neurons, damaged endothelial cells, and activated glial cells. This microenvironment produces various proinflammatory mediators (Hasturk et al., 2009), such as HMGB1, TNF-α, IL-1β, and IL-6. As HMGB1 plays an active role in the inflammatory response as an endogenous alarm, many studies have focused on HMGB1 as a cytokine that can exert therapeutic potential by inhibiting its gene expression or extracellular activity (Kikuchi et al., 2011; Andersson et al., 2018). In this study, 7 studies (Yang et al., 2013; Zhang et al., 2014; Kang et al., 2015; Bi et al., 2017; Sun et al., 2019; Wu et al., 2021) inhibited the expression of HMGB1 to varying degrees by five methods, including GL, EP, SHI, HG and HBO, among which EP, perhaps the most effective method, significantly reduced HMGB1 expression on the first day after SCI compared with the control group. Shi, GL and HG downregulated the expression of HMGB1 to varying degrees, but the overall effect was inferior to that of EP. In addition, HBO is not as effective as the other methods mentioned above, but it can also effectively inhibit HMGB1 expression. In sterile inflammation, such as traumatic spinal cord injury and ischemia–reperfusion injury, HMGB1 is considered to be an early mediator of inflammation (Chen et al., 2011). HMGB1 translocates from the nucleus to the cytoplasm after SCI, and its expression increases earlier than that of pro-inflammatory cytokines such as TNF-α, IL-1β, and IL-6. By inhibiting HMGB1 expression after spinal cord injury, its role as a proinflammatory mediator is also blocked. Therefore, in seven studies measuring TNF-α levels (Zhang et al., 2014; Kang et al., 2015; Bi et al., 2017; Nakajo et al., 2019; Fan et al., 2020; Chen et al., 2021; Wu et al., 2021), five different methods, including anti-HMGB1 Ab, GL, SHI, HG, and HBO, significantly reduced TNF-α levels. Early intervention with HMGB1 may have a positive effect on reducing the proinflammatory cascade in the early stages of SCI.

Spinal cord edema, a hallmark of spinal cord injury, aggravates primary injury by increasing intrathecal pressure, leading to bleeding and BSCB destruction and causing further injury, which triggers further cell necrosis (Ahuja et al., 2017; Quadri et al., 2020). It is mainly due to inflammation after SCI. HMGB1 is an inflammatory cytokine that is closely related to spinal cord edema after SCI (Sun et al., 2019). Studies have shown that anti-HMGB1 treatment can attenuate CNS edema and reduce the inflammatory response induced by HMGB1 (Nosaka et al., 2018; Xia et al., 2020). In five studies (Bi et al., 2017; Uezono et al., 2018; Nakajo et al., 2019; Sun et al., 2019), HMGB1 was inhibited by Shi, anti-HMGB1 Ab, EP and GL, and spinal cord water content was measured. The results showed that inhibition of HMGB1 expression reduced spinal cord water content to varying degrees. In the spinal cord, inhibition or downregulation of AQP4 overexpression leads to an attenuation in spinal cord edema after spinal cord injury (Ge et al., 2013; Hu et al., 2015). Sun et al. (2019) reported that AQP-4 expression was also significantly downregulated after inhibition of HMGB1, suggesting that inhibition of HMGB1 expression and further inhibition of edema may play a role by reducing AQP-4 overexpression.

Apoptosis is a naturally occurring physiological process and plays a key role in secondary spinal cord injury (Beattie et al., 2000; Shi et al., 2021). Finding a method to inhibit apoptosis after spinal cord injury may have important clinical significance for further treatment. HMGB1 can be passively released by necrotic neurons or damaged cells after SCI. It has been reported that the ability of Hmgb1(-/-) necrotic cells to promote inflammation was greatly reduced, which proved that the release of Hmgb1 can signal the demise of a cell to neighboring cells (Scaffidi et al., 2002). In recent years, an increasing number of studies have reported that HMGB1 plays an important role in the process of CNS apoptosis (Guo et al., 2019b; Wang and Zhang, 2020). Three studies (Bi et al., 2017; Uezono et al., 2018; Nakajo et al., 2019) in this study investigated the effect of inhibiting HMGB1 expression on apoptosis after SCI. The results showed that inhibition of HMGB1 expression by Shi and anti-HMGB1 Ab could significantly reduce the number of apoptotic cells.

NF-κB is an important inflammatory transcription factor in the CNS, that regulates many genes and signaling pathways involved in inflammation (Ma and Hottiger, 2016). Previous reports have shown that NF-κB upregulates proinflammatory cytokines, including TNF-α, IL-1β, and IL-6 in SCI (Sun et al., 2017; Liu et al., 2019). Extracellular HMGB1 activates inflammatory cells by activating the receptor for RAGE, TLR-2 and TLR-4 (van Beijnum et al., 2008; Paudel et al., 2018). Activation of any of these receptors enhances the NF-κB mediated transcription of inflammatory mediators (Kawai and Akira, 2007; Tóbon-Velasco et al., 2014). Seven studies (Yang et al., 2013; Kang et al., 2015; Bi et al., 2017; Sun et al., 2019; Fan et al., 2020; Chen et al., 2021) demonstrated that HMGB1-targeted therapy alleviated secondary injury via the NF-κB signaling pathway, including four studies (Kang et al., 2015; Bi et al., 2017; Sun et al., 2019) via the HMGB1/TLR-4/NF-κB pathway and one study (Fan et al., 2020) via the HMGB1/RAGE/NF-κB pathway. However, only one (Chen et al., 2021) of these seven studies used anti-HMGB1 Ab, so whether anti-HMGB1 Ab plays a role through the NF-κB signaling pathway needs further research.

Anti-HMGB1 therapy can jointly alleviate secondary injury after SCI through different mechanisms, and different therapeutic methods have different effects.Anti-HMGB1 Ab has been reported to be beneficial in animal models for the treatment of various types of inflammatory diseases, particularly in the CNS (Vijayakumar et al., 2019). In this study, considering the above effects of HMGB1 in rat or mouse models of TSCI, HMGB1-targeted therapy may be an effective treatment strategy after TSCI, and it improves locomotor function recovery, reduces inflammation, attenuates edema, and reduces apoptosis after TSCI. Intrathecal injection of anti-HMGB1 Ab 0-3 h after TSCI may be the most efficacious treatment. In addition, some novel therapies, such as the combination of anti-HMGB1 antibodies with other therapies, may generate more significant therapeutic effects. With the application of new therapeutic methods, we can expect the prospect of HMGB1-targeted therapy in the treatment of TSCI to improve.

### Limitations

This systematic review has several limitations. First, among the 12 articles included, only 4 articles were from Japan and the US, and the rest were all from China. The limited number of articles was also a major limitation of this review. Moreover, given the existence of publication bias, the conclusions drawn thus far may also be overestimated. In addition, according to the information reported in articles, we were unable to assess whether all baseline features were balanced among groups, and the animal models and species used in the study were different, as well as simple data integration, which also contributed to the high heterogeneity of this meta-analysis. Sex as a biological variable is an important reference factor. Male and female animals respond differently to injury. However, sex was not reported comprehensively in the included studies, and sex characteristics were not reported in the two studies, which also leads to bias. The wide variety of drug or anti-HMGB1 Ab doses, different treatment initiation times, different routes of administration, and varied methodological quality of studies could also bring confounding factors. The BBB scale or BMS scale is the most common method used to assess locomotor function recovery effects after SCI. However, it depends on the judgment and interpretation of the observer and is prone to bias.

Moreover, although all animal studies in this systematic review evaluated models of spinal cord injury in the chest, 50% of human spinal cord injuries affect the neck region, with C5 being the most commonly affected region (Alizadeh et al., 2019). There are significant anatomical differences in the spinal cord between the neck and thoracic vertebrae that affect the degree of injury and recovery.

## Conclusion

HMGB1-targeted therapy improves locomotor function recovery, reduces inflammation, attenuates edema, and reduces apoptosis in rat and mouse models of TSCI. Intrathecal injection of anti-HMGB1 Ab 0-3 h after SCI may be the most efficacious treatment. The use of this meta-analysis was limited by the poor methodological quality of the included studies. Therefore, more high-quality studies and objective evidence are needed to support preclinical HMGB1-targeted therapy for SCI.

## Data availability statement

The original contributions presented in the study are included in the article/supplementary material, further inquiries can be directed to the corresponding author.

## Author contributions

CD and LS conceived the review. CD and LD performed the literature searches, study selection, data extraction, quality assessment, and undertook the review analyses with JL. LS checked as third advisers and critically revised the manuscript. CD wrote the initial draft. All authors approved the final version of the paper.

## Funding

This study was supported by grants from the National Natural Science Foundation of China (No. 81870976) and Shanxi Provincial Health Commission Key Projects to Tackle Key Problems (No. 2020XM27).

## Conflict of interest

The authors declare that the research was conducted in the absence of any commercial or financial relationships that could be construed as a potential conflict of interest.

## Publisher's note

All claims expressed in this article are solely those of the authors and do not necessarily represent those of their affiliated organizations, or those of the publisher, the editors and the reviewers. Any product that may be evaluated in this article, or claim that may be made by its manufacturer, is not guaranteed or endorsed by the publisher.
